# Comments on: Chari, R.; Burke, T.A.; White, R.H.; Fox, M.A. Integrating Susceptibility into Environmental Policy: An Analysis of the National Ambient Air Quality Standard for Lead. *Int. J. Environ. Res. Public Health* 2012, *9*, 1077-1096

**DOI:** 10.3390/ijerph10020712

**Published:** 2013-02-08

**Authors:** Deirdre L. Murphy, Molini Patel, Ellen Kirrane, Lisa Vinikoor-Imler

**Affiliations:** United States Environmental Protection Agency, Research Triangle Park, NC 27711, USA; E-Mails: patel.molini@epa.gov (M.P.); kirrane.ellen@epa.gov (E.K.); vinikoor-imler.lisa@epa.gov (L.V.-I.)

In their recent article [1], Chari *et al.* call attention to the important subject of setting National Ambient Air Quality Standards (NAAQS) to provide requisite protection for public health, including the health of sensitive groups, as specified under the Clean Air Act (73 FR 66965) [2]. The authors focus on consideration of susceptibility to inform policy choices, using lead (Pb)-related neurocognitive effects and children from low socioeconomic status (SES) families in the context of alternative Pb standard levels. Our comments focus on the authors’ analysis of the scientific evidence and not on policy. We agree with the authors that the health effects evidence for Pb indicates a role (or roles) for SES-related factors in influencing childhood Pb exposure and associated health effects. We disagree, however, with the authors’ interpretation of the literature on SES influence on the shape of the concentration-response (C-R) relationship between children’s blood Pb and IQ (e.g., steepness of the slope). We further address aspects of the scientific evidence that are important to the consideration of sensitive populations in the context of the Pb NAAQS, and how the U.S. Environmental Protection Agency (EPA) considered this evidence in setting the Pb NAAQS in 2008. 

The role of SES as a confounder and/or effect modifier of the associations between Pb exposure and health effects is complicated [3,4]. Lower SES is independently associated with an adverse impact on neurocognitive development [5], and often associated with higher Pb exposure and higher blood Pb concentration [3]). Consequently, SES is commonly treated as a potential confounder. Several studies have, however, examined SES as a potential modifier of the association of childhood Pb exposure with cognitive function, and thus, might inform the question of a likelihood for greater neurocognitive impact at the same blood Pb level in low as compared to higher SES groups. However, this limited dataset has produced somewhat mixed results. For example, an analysis of the Port Pirie longitudinal cohort in early adolescence suggested a steeper slope for the C-R relationship for the relatively lower SES subset of the cohort, although the results lacked statistical significance after adjustment for covariates and an analysis of the same cohort in early childhood found no significant SES modification [6,7]. Comparison of results between two longitudinal study cohorts of notably different SES but similar blood Pb levels (Rochester and Boston), however, does not indicate appreciably different slopes for their blood Pb-IQ C-R relationships [8,9,10]. Additionally, a meta-analysis of data from these and other studies, which examined the relationship of early childhood blood Pb with school age IQ, observed a smaller IQ reduction per unit blood Pb for studies conducted in SES disadvantaged populations compared to nondisadvantaged populations [11]. In summary, we find, based on a critical assessment of the available scientific evidence, that low SES is not consistently associated with a larger decrement in IQ per increment of children’s blood Pb level. 

Building from their interpretation of the evidence on SES as a Pb susceptibility factor, Chari *et al.* [1] present an alternative application of the evidence-based framework that was developed by the EPA to inform the 2008 Pb NAAQS. The authors suggest that their application, which involved their use of different C-R functions or slopes of the C-R relationship between children’s blood Pb and IQ than those selected by EPA, incorporates susceptibility, and that EPA’s application did not. As discussed below, however, EPA comprehensively considered multiple aspects of the evidence relating to susceptibility.

Based on the extensive evidence base for the health effects of Pb, including the evidence pertaining to sensitive populations, EPA focused their consideration for a Pb NAAQS level primarily on Pb effects to the developing nervous system in children (73 FR 66965) [2,3]. (EPA’s assessment of the extensive evidence base for the health effects of Pb, including the evidence pertaining to sensitive populations, was documented in the Air Quality Criteria for Pb [3], two drafts of which received public scientific review by the Clean Air Scientific Advisory Committee (CASAC), supplemented by subject-matter-expert Panelists, creating a scientific panel of 20. Among the range of health effects aspects considered by the CASAC review was identification of populations that are especially susceptible or vulnerable to Pb.) EPA further gave particular attention to neurocognitive effects at the blood Pb levels well below 10 µg/dL that have become common in U.S. children, the shape of the C-R relationship at those blood Pb levels, and available evidence regarding factors affecting this relationship. Thus, an important factor in the development and application of the evidence-based framework to inform EPA’s NAAQS decision was the conclusion of a steeper slope to the C-R relationship for children’s blood Pb level and IQ at lower (*versus* higher) blood Pb levels [3]. In light of this conclusion and the recognition that much of the evidence comprised studies involving blood Pb levels much higher than those common in U.S. children, EPA focused on studies involving the lowest studied blood Pb levels, closest to those of today’s U.S. children. In so doing, EPA selected four C-R relationship slopes from four studies with subgroup analyses for which mean blood Pb levels ranged from 2.9 to 3.8 µg/dL [8,10,12,13]. Consistent with the conclusion regarding a steeper slope at lower blood Pb levels, these studies were those reporting the steepest slopes in the evidence base. (Among studies with mean blood Pb levels near or below 10 µg/dL, EPA considered slopes from published analyses which found statistically significant associations of neurocognitive decrement with blood Pb after adjustment for important potential confounders (or their surrogates) in full study cohorts, as well as some subgroups restricted to children with lower blood Pb levels (73 FR 66977) [2].) Thus, with the selection of these slopes, EPA focused this aspect of the framework on the evidence pertaining to those children with the greatest incremental impact of Pb on IQ. The rest of the framework design, which implemented relationships between air Pb and blood Pb for different standard levels, further focused EPA’s decision on the level for a revised standard on the sensitive subset of children in the U.S. likely to be exposed at different alternative standard levels, which is generally expected to be the subset living near sources who are likely to be most highly exposed to air Pb (73 FR 67005) [2]. 

With regard to Chari and colleague’s consideration of EPA’s evidence-based framework, we first note, that it appears they did not realize the studies selected by EPA did not represent SES-diverse populations. Three of the four analyses from which EPA identified C-R relationship slopes (C-R functions) for use in the evidence-based framework are for subgroup analyses of study groups completely or largely composed of children from families of low or low to middle income [8,12,13]. The fourth slope, for a subgroup of the middle to high SES Boston longitudinal cohort [10], is not appreciably different from the slopes obtained for the three lower SES datasets. 

We further note that the C-R relationship slopes identified in Table 3 of Chari *et al.* [1] as representing analyses for low SES children were derived by extrapolation, using log-linear functions in all but one case, to a blood Pb level of 2 µg/dL, a value largely outside the range of the study data. For example, all but one of the slopes are based on studies in which the mean blood Pb levels for the full cohorts exceed 14 µg/dL [6,14,15]. (The fourth slope comes from analyses of neonatal blood Pb, reflecting maternal exposure rather than childhood exposure (relevant for the evidence-based framework), with the full neonate cohort mean level being 4.5 µg/dL [16]. Further, at least two of the studies reported there to be still higher blood Pb levels in the lower SES subgroup than the full cohort [6,16].) The use of extrapolation, particularly for the nonlinear functions, adds uncertainty and reduces confidence in the estimates. In contrast, the slopes used by EPA in the evidence-based framework reflected linear functions derived directly from the four study subgroup datasets without extrapolation (73 FR Tables 1 and 3) [2].

In summary, there is agreement that evaluation of differential susceptibility is important to inform policy choices and the full evidence base indicates that SES-related factors are important influences on Pb exposure and on children’s cognitive function. A critical assessment of the evidence on children’s blood Pb and IQ, however, does not support the underlying premise of the presentation by Chari *et al.* of a larger incremental effect in low (as compared to higher) SES populations. Additional research in this area may further inform this issue.
